# Testing a pyriproxyfen auto-dissemination station attractive to gravid *Anopheles gambiae sensu stricto* for the development of a novel attract-release -and-kill strategy for malaria vector control

**DOI:** 10.1186/s12879-019-4438-9

**Published:** 2019-09-11

**Authors:** Oscar Mbare, Steven W. Lindsay, Ulrike Fillinger

**Affiliations:** 1 International Centre of Insect Physiology and Ecology, Human Health Theme, Nairobi, Kenya; 2 London School of Hygiene and Tropical Medicine, London, UK; 3 Durham University, Durham, UK

**Keywords:** *Anopheles gambiae* sensu stricto, Pyriproxyfen, Oviposition, Vector control

## Abstract

**Background:**

Larviciding is an effective supplementary tool for malaria vector control, but the identification and accessibility of aquatic habitats impedes application. Dissemination of the insect growth regulator, pyriproxyfen (PPF), by gravid *Anopheles* might constitute a novel application strategy. This study aimed to explore the feasibility of using an attractive bait-station to contaminate gravid *Anopheles gambiae* sensu stricto with PPF and subsequently transfer PPF to larval habitats.

**Methods:**

A bait-station was developed comprising of an artificial pond containing water treated with 20 ppm cedrol, an oviposition attractant, and a netting-cover treated with PPF. Three identical semi-field cages were used to assess the potential of gravid *Anopheles* to transfer PPF from the bait-station to ponds. Gravid females were released in two semi-field cages, one with PPF on its bait-station (test) and one without PPF (control). No mosquitoes were released in the third cage with a PPF-treated station (control). Transfer of PPF to open ponds was assessed by monitoring emergence of late instar insectary-reared larvae introduced into the ponds. The amount of PPF carried by a mosquito and transferred to water was quantified using liquid chromatography-mass spectrometry.

**Results:**

In the controls, 86% (95% CI 81–89%) of larvae introduced into open ponds developed into adults, indicating that wind did not distribute PPF in absence of mosquitoes. Emergence inhibition was observed in the test cage but was dependent on the distance between pond and bait-station. Only 25% (95% CI 22–29%) of larvae emerged as adults from ponds 4 m from the bait-station, but 92% (95% CI 89–94%) emerged from ponds 10 m away. Each mosquito was contaminated on average with 112 μg (95% CI 93–123 μg) PPF resulting in the transfer of 230 ng/L (95% CI 180–290 ng/L) PPF to 100 ml volumes of water.

**Conclusions:**

The bait-stations successfully attracted gravid females which were subsequently dusted with effective levels of PPF. However, in this study design, attraction and dissemination was limited to short distances. To make this approach feasible for malaria vector control, stronger attractants that lure gravid females from longer distances, in landscapes with many water bodies, and better PPF delivery systems are needed.

## Background

Improved access to the core malaria control interventions namely vector control, effective diagnosis and prompt treatment have greatly contributed to the global reduction in malaria morbidity and mortality [1–3]. However, recent World Malaria Reports from 2017 and 2018 indicate that this remarkable progress has stalled [1, 4]. This worrying trend emphasizes the need to explore additional tools for malaria prevention to supplement the current frontline measures and ensure that the gains achieved in the last decade are sustained [5–7].

Malaria control programs are encouraged to adopt integrated vector management strategies to increase efficacy, cost-effectiveness and sustainability of disease control [7, 8]. Larval source management (LSM) targeting immature mosquito vectors in their aquatic habitats, such as larviciding and environmental management, can effectively serve as supplementary vector control measures [9, 10]. Studies in East Africa highlighted the benefit of combining long-lasting insecticidal nets (LLINs) and larviciding with microbial larvicides for reducing transmission [11–14]. However, the challenge associated specifically with larviciding is the need to reach all available and potentially suitable aquatic habitats in an intervention area [15–18], many of which might only be reached by aerial application. Whilst it has been suggested that larviciding might be targeted only at a proportion of most productive habitats [19, 20], habitat productivity is still poorly understood and not easily predicted by application teams [21–23].

Auto-dissemination, a novel strategy that exploits the adult insect as a ‘vehicle’ to deliver insecticide, might be one way of addressing this challenge. This strategy has been shown to be effective in the control of *Aedes* mosquito [24–27], leading to an increased interest in its exploration for control of Afrotropical malaria vectors [28, 29]. Semi-field studies conducted in Tanzania provide the first evidence of the potential of *An. arabiensis* to transfer the insecticide pyriproxyfen (PPF) from resting surfaces to larval habitats, consequently inhibiting larval development [30–32]. However, these studies were implemented at extremely high mosquito population densities that are unlikely to occur under natural conditions. Furthermore, the targeting of host-seeking mosquitoes before or shortly after bloodmeals for contact with PPF is likely to cause a large proportion of the females to become sterilized and not develop into gravid females [33–37]. We recently showed that a female that is not gravid is significantly less likely to visit an oviposition habitat, and hence transfer PPF to a water body, and that the optimum time for exposing female *Anopheles gambiae* sensu stricto to PPF for auto-dissemination is close to oviposition [35]. Consequently, the aim of this study was to design an attractive bait-station to contaminate gravid *An. gambiae s.s.* with PPF and to test the efficiency of PPF transfer to open ponds under semi-field conditions.

## Methods

### Study site

Experiments were carried out in large netting-screened semi-field cages (10.8 m long × 6.7 m wide × 2.4 m high) at the International Centre of Insect Physiology and Ecology, Thomas Odhiambo Campus (*icipe*-TOC), located on the shore of Lake Victoria in Mbita, Homa Bay county, western Kenya (geographic coordinates 0^0^ 26′ 06.19″ S, 34^0^ 12′ 53.13″ E; altitude 1137 m above sea level). The cages had a sand floor and did not contain any vegetation. Mbita is characterized by tropical climate with an annual average minimum temperature of 16 °C and maximum temperature of 29 °C. The area experiences two rainy seasons; the long rains between March and June and the short rains between October and December.

### Test insecticide

An experimental formulation of dust, with particles 12 μm diameter, containing 10% of pyriproxyfen (PPF) (Sumilarv®, Sumitomo Chemical Company) was used in all experiments.

### Mosquitoes

*Anopheles gambiae s.s.* (Mbita strain) larvae and adults used in this study were obtained from the mosquito insectaries at *icipe*-TOC. Immature stages were reared in a semi-field cage at ambient conditions with average daily temperature of 25–28 °C, relative humidity of 68–75% and natural lighting. Mosquito larvae were reared in round plastic tubs (diameter 60 cm) filled with 5 L water (5 cm deep) from Lake Victoria filtered through a charcoal-sand filter. Mosquito larvae were fed with a pinch of fish food (Tetramin©Baby) twice daily. Third (late) instar mosquito larvae were randomly selected from different tubs to ensure that cohorts of larvae used in experiments were a representative sample of the size distribution of the experimental larval population. Adult mosquitoes were held in mosquito-netting covered cages (30 cm × 30 cm × 30 cm) in a holding room with ambient climate conditions and provided with 6% glucose solution ad libitum. Three-day old females were allowed to feed on a human arm on two consecutive nights. Gravid mosquitoes were used for experiments in this study.

### Development of a bait-station

#### Contamination of adult *An. gambiae s.s.* with PPF

Water vapour has been shown to attract gravid malaria vectors [38] and hence it was considered essential to include water in the bait-station. Females were prevented from accessing the water to lay eggs using fly gauze (black fibre-glass netting gauze, mesh size 1 mm × 1 mm). To determine the best method to treat netting surfaces with PPF for efficient contamination of mosquitoes, preliminary cage tests were conducted in small-sized cages (30 cm × 30 cm × 30 cm). Two methods of applying PPF on the netting gauze that served as the dissemination platforms were compared. First, the netting gauze (diameter 7 cm) was treated with 1 g of PPF dust applied with a soft brush to ensure uniform spreading of the PPF over the netting surface. Second, 1 g of PPF dust was mixed with 2 ml of cooking oil and applied to the netting gauze with a soft brush and left to dry in the air for 30 min. The rationale for this was to test a formulation that would be easier to apply and less likely to be distributed by wind.

Each experimental cage was provided with two glass cups (Pyrex®, capacity 100 ml, diameter 7 cm) and the cups were placed at the diagonal corners of the cage, approximately 26 cm apart. The first cup in each cage was filled with 100 ml non-chlorinated tap water and left open for gravid mosquitoes to lay eggs. The second cup, serving as the bait-station, was filled with six-day old soil infusion previously shown to attract gravid *An. gambiae s.s* [39]. Soil infusion was prepared by incubating 15 L of non-chlorinated tap water with 2 kg of soil collected from a known breeding habitat of *An. gambiae* sensu *lato* [39] which was dry at the time of collection. Infusions were prepared in round plastic tubs (diameter 0.42 m) and left for six days before use in experiments as described in detail previously [39]. The top of the bait-station in the control cages was covered with untreated netting gauze while in the test cages it was covered with netting gauze treated with either PPF dust or PPF dust formulated in oil. In each cage five gravid *An. gambiae s.s.* were released at 18:00 h and left overnight. The following morning, open oviposition cups were assessed for the presence of eggs. To confirm the transfer of PPF in test cages, 10 insectary-reared late instar *An. gambiae s.s.* larvae were introduced into oviposition cups in all cages and monitored for adult emergence. Introduced larvae were fed daily on a pinch of Tetramin®Baby fish food. Pupae that developed were transferred with a small volume of water from the oviposition cup into plastic cups (diameter 7 cm) and monitored for adult emergence. It took 6–7 days for all larvae introduced into the oviposition cups to develop into adults or die. These experiments were conducted over three rounds on separate dates. There were five replicate cages per treatment in each experimental round, thus in total there were 15 cages with untreated bait-stations, 15 cages with bait-stations treated with PPF dust and 15 cages with bait-stations treated with PPF dust formulated in oil. Oviposition cups were randomly allocated to one of the four corners in the cages.

#### Luring gravid *An. gambiae s.s.* to a pond

**Fig. 1:**
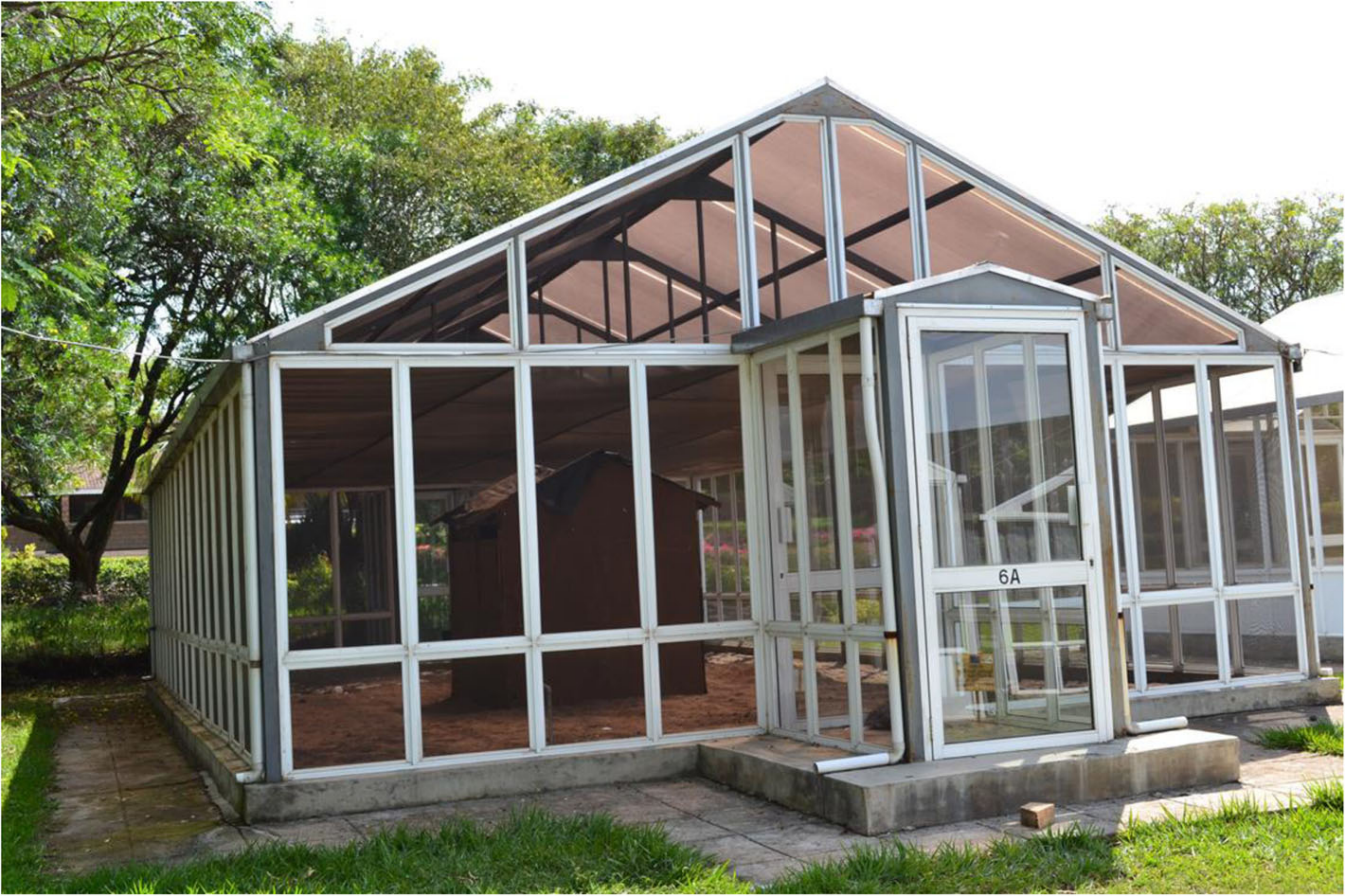
Semi-field cage showing artificial hut constructed at the centre of the semi-field cage

Preliminary experiments were conducted in a semi-field cage (Fig. 1) to compare attractiveness of two substrates that attract gravid *An. gambiae s.s*.: a six-day old soil-infusion [39] and the sesquiterpene alcohol, cedrol (Cedrol ≥99.0% (sum of enantiomers, GC, Sigma-Aldrich, Steinheim, USA) [40]. The two substrates were evaluated separately on different dates. Four artificial ponds were created by digging holes in the sand at the four corners of the cage, and each hole was filled with a round enamel tub (diameter 0.42 m, depth 8 cm). The tubs were placed 1 m away from the nearest wall. During each experimental round, three of the tubs were filled with 7 L of non-chlorinated tap water as oviposition habitats while the fourth tub was filled with a test substrate to attract gravid females introduced into the cage. The test substrate consisted of either 7 L of six-day old soil infusion or 7 L of non-chlorinated tap water treated with cedrol. Two concentrations of cedrol were tested sequentially: 5 ppm and 20 ppm. Cedrol was prepared in ethanol by first preparing a stock solution of 10,000 ppm by dissolving 150 mg of cedrol to 15 ml of absolute ethanol (≥99.8% (GC), Sigma Aldrich). Dilutions were made by adding the appropriate volume of stock solution to water in the pond. For instance, 5 ppm cedrol was prepared by adding 3.5 ml of stock solution into 7 L of water. Similarly, 20 ppm cedrol was prepared by adding 14 ml of stock solution to the 7 L of water in the artificial pond.

A small wooden hut (1.78 m long × 1.73 wide × 1.80 m high) was set up in the centre of the semi-field cage (Fig. 1) to simulate the natural indoor environment where female *An. gambiae s.s.* take a blood-meal and rest till they are gravid [41]. The hut had a door and two windows that were shut when the experiment was in progress. Two open eaves (1.70 m × 0.18 m) located at opposite sides of the hut served as the only exit points for the mosquitoes released in the hut. In each experimental night 200 gravid *An. gambiae s.s.* were released at 18:00 h inside the hut.

To measure the number of mosquitoes visiting the ponds, the top of each pond was covered by a black fibre-glass netting gauze cut to size (diameter 0.42 m) on which a fine film of insect glue was sprayed (Oeco insect spray, Oecos, UK) to trap the gravid females as they searched for oviposition substrates. At 6:00 h the following morning the number of females trapped on the sticky screens was counted. Each of the test substrates was evaluated over 12 replicate nights with fresh oviposition substrates and fresh batches of mosquitoes used each night. The four ponds (three ponds filled with non-chlorinated water and the pond containing the test substrate) were randomly allocated in all four corners of the semi-field cage using a randomized complete block design.

### Evaluation of the auto-dissemination of PPF by gravid *An. gambiae s.s.* from the bait-station to larval habitats

**Fig. 2:**
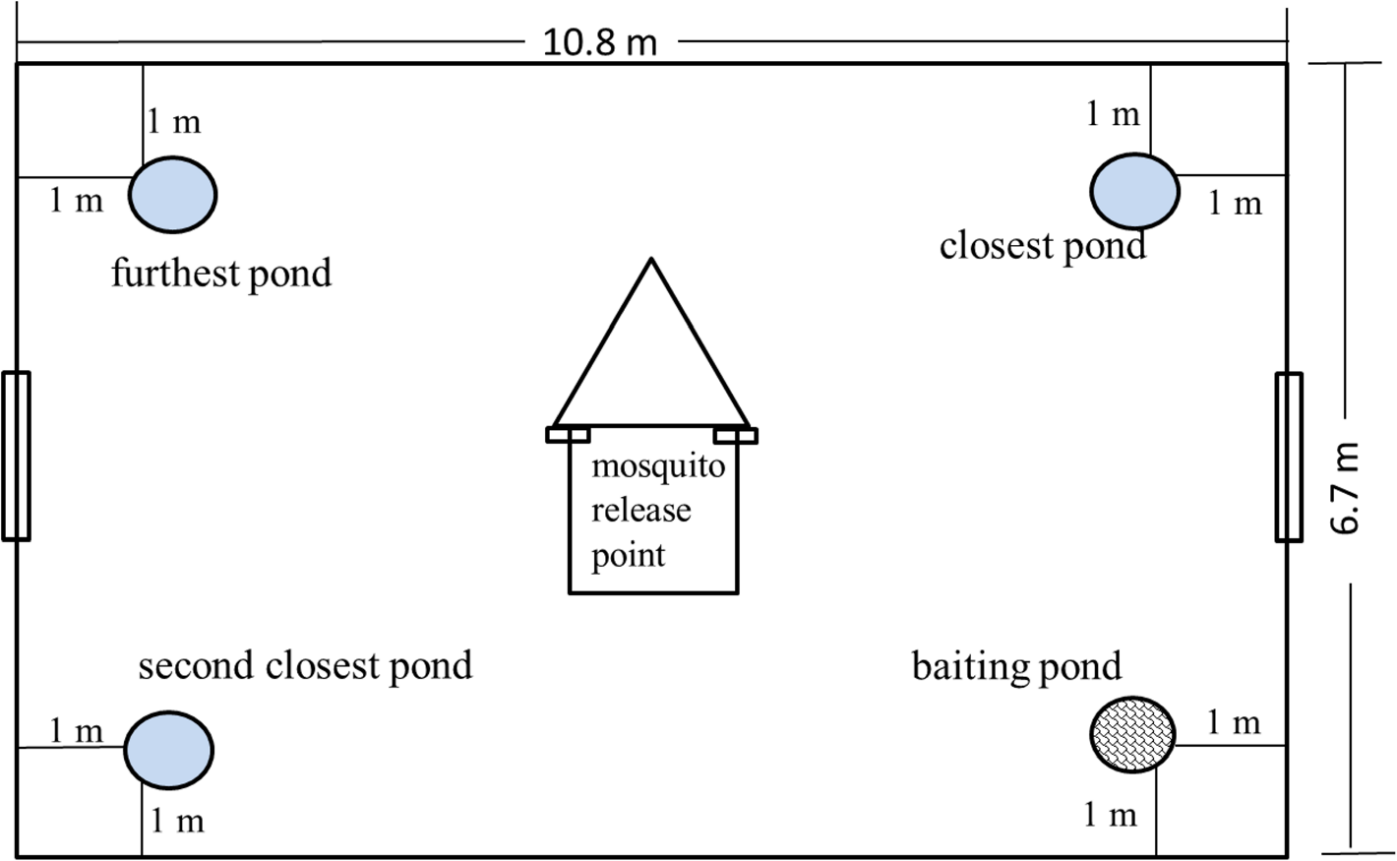
Schematic representation of semi-field cage showing location of ponds and the artificial hut that serve as release point of gravid mosquitoes

These experiments were conducted in three identical semi-field cages which contained a wooden hut at the centre and four enamel tubs used to create artificial ponds at the corners of each cage as described above (Fig. 2). The experiments were done under standardized conditions without vegetation. In the first semi-field cage, three ponds were filled with 7 L of non-chlorinated tap water each and left open for mosquito oviposition, while the fourth pond serving as the bait-station contained 7 L of water treated with 20 ppm cedrol as described above. A netting gauze (diameter 0.42 m) treated with 3.5 g PPF dust (average 20.3 g PPF/m^2^ retained on gauze on weighing) was placed on top of the cedrol-treated pond. The three open ponds were 4.4 m, 8.4 m and 10.3 m away from the bait-station (Fig. 2). The set-up in the second semi-field cage was the same as the first, except that no mosquitoes were released in the cage. The aim here was to investigate if PPF might be distributed by air movement to neighbouring ponds, rather than mosquitoes. In the third semi-field cage, mosquitoes were released but the netting gauze placed on top of the bait-station was left untreated. This set-up served to investigate natural adult mosquito emergence rates from ponds when no insecticide was present in the semi-field cage. Two hundred gravid *An. gambiae s.s.* were released at 18.00 h per experimental night inside the hut and allowed to disperse through the open eaves.

The following morning, all open ponds in the three semi-field cages were visually assessed for the presence of eggs laid. Eggs were not counted since an exact estimate would have required removing the eggs from the ponds using a sieve or similar tools potentially interfering with the amount of PPF transferred. To ensure sufficient replication of the experiment, the impact of PPF was not assessed by monitoring the development of eggs laid in ponds by exposed mosquitoes. That would have taken approximately two weeks to complete per replicate and, therefore, over half a year to complete 12 replicates [42]. Instead, the possible transfer of PPF by females to the ponds was assessed by monitoring the adult emergence of 50 insectary-reared late instar *An. gambiae s.s.* larvae that were introduced into each open pond in all three experimental set-ups. The larvae were introduced into the ponds in the morning after the gravid females were released. Introduced larvae were fed daily with a pinch of Tetramin®Baby Fish food. Any pupae that developed in the three ponds were transferred with a small volume of water from the pond into 200 ml plastic cups (diameter 7 cm) and monitored for adult emergence. It took 6–7 days for all introduced larvae to develop into adults or die. Thereafter the ponds and hut were cleaned and all remaining flying adult mosquitoes in cages aspirated using a motorized backpack aspirator (John W. Hock Company, USA). A new round of replicates was set-up with fresh oviposition substrates and fresh batches of gravid mosquitoes and mosquito larvae. The experiment was replicated over 12 rounds with each round lasting seven days. The four ponds were randomly allocated in all four corners of the three semi-field cages in a randomized complete block design. To avoid contamination, specific semi-field cages were dedicated to the test and controls.

### Liquid-chromatography-mass spectrometry (LC-MS) quantification of the amount of PPF carried by an individual mosquito and transferred to a water sample

An enamel bowl (diameter 0.42 m) filled with 7 L of non-chlorinated tap water was introduced into a 60 × 60 × 60 cm cage (BugDorm-2120F; MegaView Science Taiwan). The top of the bowl was covered with black fibre-glass netting gauze treated with 3.5 g PPF dust (average 20.3 g PPF/m^2^ retained on gauze on weighing) as described above. Gravid *An. gambiae s.s*. were introduced into the cage and monitored for contact with the PPF-treated netting gauze. At any time, there were only two females in the cage. Females that contacted the PPF-treated netting material at least twice were gently aspirated from the cage into holding containers.

Two different tests were conducted with females that contacted the PPF-treated netting. First, 200 potentially contaminated females were individually transferred into 1.5 ml Eppendorf tubes and frozen at − 70 °C until they were used to quantify the amount of PPF on their body. Secondly, 30 potentially contaminated females were used to determine the amount of PPF that a single mosquito transfers to water during oviposition. For this, bioassays were conducted by introducing these females individually into 15 cm × 15 cm × 15 cm cages provided with a glass cup (Pyrex®, 100 ml, diameter 7 cm) filled with 100 ml of non-chlorinated tap water. The females were left overnight in the cages to lay eggs. The following morning the glass cups were assessed for presence of eggs laid.

To confirm the transfer of PPF into the oviposition water, 10 insectary-reared late instar *An. gambiae s.s.* larvae were introduced into all cups in which females had laid eggs and monitored for adult emergence as described above. When all introduced larvae had died or emerged as adults, the water from the cups was transferred into 50 ml glass jars. The water samples were frozen at − 70 °C awaiting chromatographic quantification of PPF in the samples. Comparisons were made to a control group of gravid females that were unexposed to PPF. Thirty replicates of test and control cages were done.

For quantification of the amount of PPF that contaminates a gravid mosquito when she makes contact with a PPF-treated netting material, PPF was washed off the body of individual mosquitoes using 1.5 ml methanol (Sigma Aldrich, 99.9% HPLC grade) in Eppendorf tubes. The content of the Eppendorf tubes was agitated in a sonicator (Branson 2510 Ultrasonic cleaner, Eagle Road, Danbury) at 25 °C for 5 min. It was then centrifuged at 13,000 rpm (rpm) for 5 min in a microcentrifuge (PRISM™). The supernatant was transferred into 2 ml glass vials and used for detection of PPF by liquid chromatography-mass spectrometry (LC-MS).

To detect PPF in water samples used in bioassays, the samples were first pooled into groups of 10 before extraction (10 × 50 ml). Thus, there were six pools of water samples in which females that contacted PPF laid eggs and another six pools of water samples in which females unexposed to PPF laid eggs. Each pool of water sample (500 ml) was extracted in 200 ml chloroform (Sigma Aldrich, 99.9% HPLC grade) to separate the aqueous and organic layers. The organic layer, where PPF was expected to dissolve, was concentrated by evaporating it to dryness in a rotary evaporator (Heidolph Instruments, Germany). The residue was dissolved in 1 ml methanol and stored at 4 °C awaiting analysis. To assist in quantification of PPF, a known concentration (0.00002 μg) of 4-benzylbiphenyl (99%, Sigma Aldrich) was added into each extracted water sample as internal standard just before the LC-MS run. The LC-MS run was performed using electron spray ionization (LC/ESI-MS). First, the standards of pure 10% PPF and 4-benzylbiphenyl were initially run separately in the LC-MS system to confirm the retention times of PPF and the internal standard. PPF used as standard was prepared by dissolving 40 mg of PPF (10%) in 1.5 ml ethanol in a 2 ml glass vial. This was agitated in a sonicator at 25 °C for 5 min. The mixture was centrifuged at 13,000 rpm for 5 min. The supernatant was transferred into 2 ml glass vials ready for detection of PPF. The peaks of PPF and 4-benzylbiphenyl at the retention times were identified based on the molecular masses of their individual ions (molecular masses of PPF-322 and 4-benzylbiphenyl-247).

The LC/ESI-MS used consisted of a quaternary LC pump (Model 1200) coupled to Agilent MSD 6120-Single quadruple MS with electrospray source (Palo Alto, CA). The MS component of the system was used to verify the peak assigned to PPF or 4-benzylbiphenyl as the active ingredients based on the identification of molecular masses of the ions. The system was controlled using ChemStation software (Hewlett-Packard). Reverse-phase liquid chromatography was performed using an Agilent Technologies 1200 infinite series LC, equipped with a Zorbax Eclipse Plus C_18_ column, 4.6 × 100 mm × 3.5 μm (Phenomenex, Torrance, CA). A gradient using A (5% formic acid in LC-grade ultra pure H_2_O) and B (LC-grade methanol) (Sigma, St. Louis, MO) was used; 0–5 min, 95–100% B; 5–10 min, 100% B; 100–5 min. The mobile phase liquid was acetonitrile (Sigma Aldrich). The flow rate was held constant at 0.7 mL min^− 1^. The sample injection volume was 100 μl, and data were acquired in a full-scan positive-ion mode using a 100 to 500 *m/z* scan range. The dwell time for each ion was 50 ms. Other parameters of the mass spectrometer were as follows: capillary voltage, 3.0 kV; cone voltage, 70 V; extract voltage, 5 V; RF voltage, 0.5 V; source temperature, 110 °C; nitrogen gas temperature for desolvation, 350 °C; and nitrogen gas flow for desolvation, 400 L/h.

### Data analysis

Data were analysed in R statistical software package version 2.13. Generalized estimating equations (GEE) were used to analyse all data with experimental round/night included as repeated measure in the models [43, 44]. Data collected in cage and semi-field experiments to determine the transfer of PPF to water were analysed as proportions. Proportions were analysed by fitting a binomial distribution with a logit function and an exchangeable correlation matrix. Preliminary cage bioassays testing the two PPF formulations were analysed by including treatment (cage with untreated bait-station, bait-station treated with PPF dust or PPF dust formulated in oil) as fixed factor with the control cage (cage with untreated bait-station) used as the reference [45]. For the analysis of the semi-field experiments, the open pond ID identified by its distance from the bait-station was used as the fixed factor with the pond closest to the bait-station used as the reference. Count data evaluating the number of mosquitoes visiting ponds treated with soil infusion or cedrol were fitted to a Poisson distribution with a log link function. Here the ponds were included in the model as fixed factors with the bait-station used as reference. All means (proportions or counts) per treatment and their corresponding 95% confidence intervals (CIs) were modelled as the exponential of the parameter estimated for the individual models with no intercept included.

## Results

### Gravid *Anopheles gambiae s.s.* pick up more PPF when the PPF is applied on the netting surface as dust than when applied as PPF formulated in oil

**Fig. 3:**
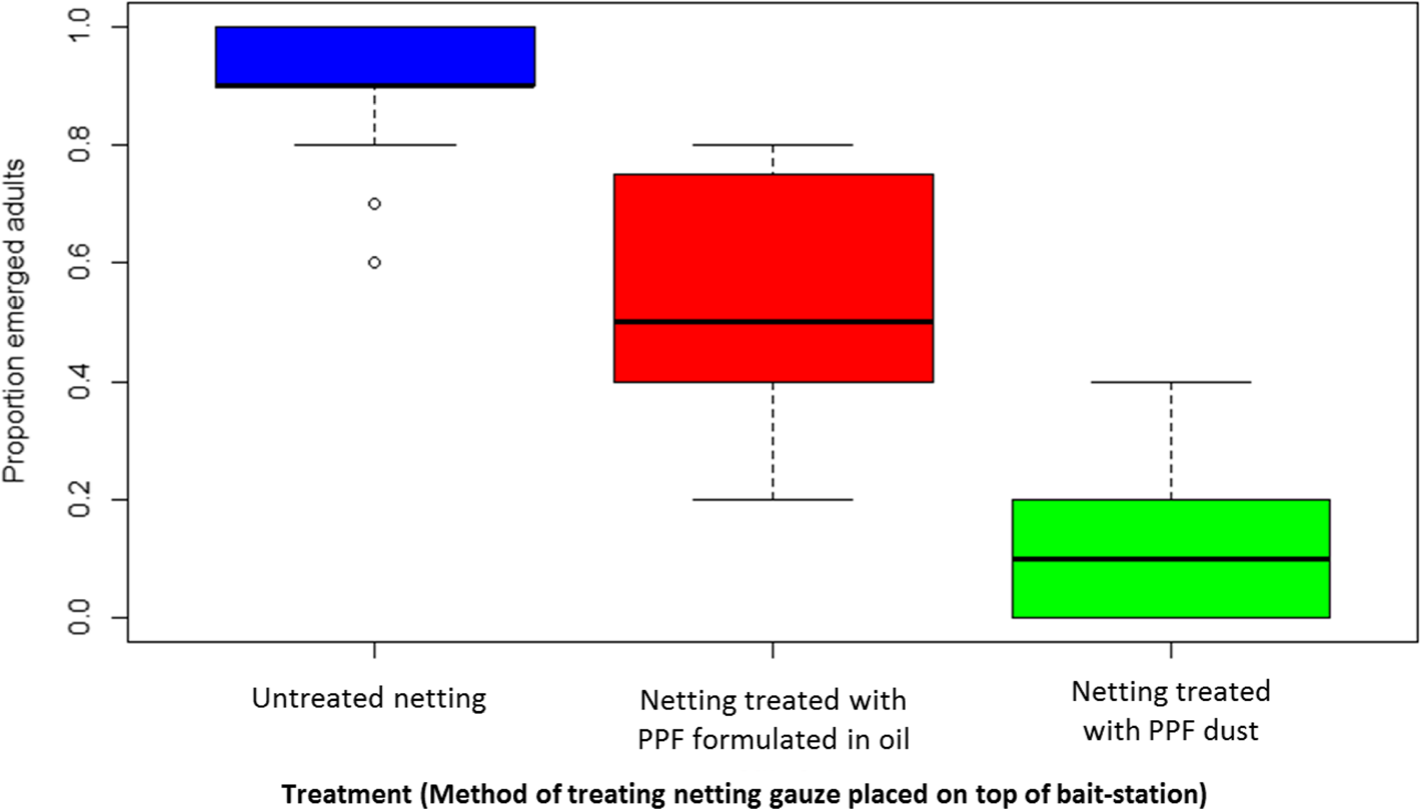
Box and whisker plots showing the median proportion and interquartile range of adult emerged in cage experiments to determine the best method to treat netting with PPF

Both methods of applying PPF on the netting gauze of the bait-station led to the transfer of PPF by females to the open oviposition cups. This is confirmed by the significant reduction in the emergence of adults from larvae introduced into oviposition cups in treatment cages with bait-station treated with PPF compared to the control cages (Fig. 3 and Table 1). However, the adult mosquito emergence rates were five times lower when the PPF was applied on the netting as dust than when it was formulated in oil.

**Table 1:** Adult emergence rates from larvae introduced into oviposition cups in cage experiments comparing PPF dust and PPF-oil applications for the bait-station

Method of PPF presentation	Mean proportion emergence (95% CI)	Odds ratio (95% CI)	*p*-value
No PPF	0.89 (0.83–0.93)	1	
Oil-formulated PPF	0.55 (0.35–0.62)	0.14 (0.07–0.28)	< 0.001
PPF dust	0.11 (0.07–0.17)	0.02 (0.006–0.036)	< 0.001

### Oviposition attractants can lure gravid *An. gambiae s.s.* to a bait-station under semi-field conditions

The number of mosquitoes trapped on the sticky screens placed over ponds containing soil infusion or cedrol at 5 ppm or 20 ppm was significantly higher than the number trapped over ponds with untreated water (Table 2). The attractiveness of soil infusion and water treated with 5 ppm cedrol was similar and not very strong; a female was only approximately 1.3 times more likely to land on the test pond than on a control pond (Table 2). However, when the water was treated with 20 ppm of cedrol, it was approximately twice as likely for a female to be trapped on the single test pond compared to any of the three control ponds in the experiment (Table 2).

**Table 2:** Semi-field oviposition choice tests evaluating the attractiveness of oviposition substrates added to water in comparison to ponds filled with water only

Treatment	Mean number of females attracted to pond (95% CI)	Rate ratio (95% CI)	*p*-value
Testing the attraction to soil infusion			
soil infusion	39 (33–45)	1	
water (pond 1)	29 (25–33)	0.74 (0.59–0.93)	0.011
water (pond 2)	26 (22–31)	0.67 (0.54–0.82)	< 0.001
water (pond 3)	27 (23–31)	0.70 (0.56–0.88)	0.002
Testing the attraction to water treated with 5 ppm cedrol			
5 ppm cedrol	33 (30–35)	1	
water (pond 1)	25 (21–29)	0.76 (0.63–0.92)	0.005
water (pond 2)	26 (23–29)	0.80 (0.70–0.91)	0.001
water (pond 3)	26 (23–30)	0.81 (0.70–0.94)	0.005
Testing the attraction to water treated with 20 ppm cedrol			
20 ppm cedrol	52 (46–60)	1	
water (pond 1)	28 (24–33)	0.54 (0.43–0.67)	< 0.001
water (pond 2)	32 (28–38)	0.62 (0.48–0.80)	< 0.001
water (pond 3)	27 (21–35)	0.52 (0.38–0.72)	< 0.001

### Transfer of PPF by gravid *An. gambiae s.s.* is dependent on the distance of the oviposition habitat from the bait-station

In all semi-field cages where gravid females were released inside the hut, eggs were observed in all open ponds on any experimental night. In the absence of PPF-treated netting gauze on the bait-station as well as in the absence of gravid females in a semi-field cage, on average 86% (95% CI 81–89%) of larvae introduced into ponds emerged as adults. For some unexplained reason there were differences in the emergence rates of larvae introduced into the ponds in the two control set-ups.

The emergence of larvae in the control experiment where no mosquitoes were released but PPF-treated netting placed over the bait-station was consistently higher than in the other control experiment where mosquitoes were released but untreated netting placed over the bait-station (Table 3). This might have been due to some microclimate differences in the two semi-field cages or an unexplained interaction between the early instars originating from eggs laid by gravid females and insectary-reared larvae introduced into ponds in one of the control semi-field cage. Importantly, in both control experiments, emergence rates were similar in all three open ponds (Table 3) and it can be excluded that wind transferred PPF from the bait-station to the open ponds.

**Table 3:** Adult emergence rates of larvae introduced into open ponds in the three experiments to evaluate transfer of PPF in semi-field cages

Ponds (distance betweeen pond and pond that served as bait station)	Mean proportion adult emergence (95% CI)	Odds ratio (95% CI)	*p*-value
Control 1 - Mosquitoes released in semi-field cage & bait-station without PPF			
closest to bait station (4.4 m)	0.85 (0.82–0.87)	1	
medium to bait-station (8.4 m)	0.83 (0.80–0.86)	0.87 (0.62–1.23)	0.443
furthest to bait-station (10.3 m)	0.84 (0.81–0.87)	0.99 (0.71–1.37)	0.944
Control 2 - No mosquitoes released in semi-field cage & bait-station with PPF			
closest to bait-station (4.4 m)	0.89 (0.86–0.91)	1	
medium to bait-station (8.4 m)	0.89 (0.87–0.92)	1.03 (0.72–1.49)	0.854
furthest to bait-station (10.3 m)	0.88 (0.85–0.91)	0.94 (0.65–1.34)	0.721
Test - Mosquitoes released in semi-field cage & bait-station with PPF			
closest to bait-station (4.4 m)	0.25 (0.22–0.29)	1	
medium to bait-station (8.4 m)	0.58 (0.54–0.62)	4.07 (3.19–5.21)	< 0.001
furthest to bait-station (10.3 m)	0.92 (0.89–0.94)	33.89 (24.16–48.47)	< 0.001
Emergence inhibition due to auto-dissemination – comparison of test with control			
control	0.89 (0.84–0.94)	1	
closest test pond (4.4 m)	0.25 (0.20–0.33)	0.042 (0.023–0.077)	< 0.001
medium test (8.4 m)	0.58 (0.51–0.66)	0.173 (0.098–0.303)	< 0.001
furthest test (10.3 m)	0.92 (0.85–0.98)	1.437 (0.846–2.444)	0.180

The presence of a PPF-treated bait-station in the test cage reduced the emergence of adults from the three open ponds confirming that PPF was transferred by the released gravid females. On average in the test cage, 25% (95% CI 22–29%) of introduced larvae emerged from the pond closest to PPF-treated dissemination station. When comparing the emergence rates between test and control experiments, significant emergence inhibition was observed for the two ponds closest to the bait-station. It was approximately 24 times less likely for an adult to emerge from the ponds closest to a bait-station (located 4.4 m away) and 6 times less likely from the ponds that were approximately twice as far away from the bait-station (located 8.4 m away) than it was for an adult to emerge from any pond in the control experiments (Table 3). No emergence inhibition was recorded from the pond that was furthest away from the bait-station, located in the opposite corner and obstructed by the hut suggesting that no or an insufficient amount of PPF was transferred to this pond.

### Gravid *An. gambiae s.s.* transfer only a small fraction of the PPF they pick up to the oviposition substrate

Ninety percent (*n* = 30) of females that landed on PPF-treated netting laid eggs when provided with water in a glass cup in a cage. A similar number (n = 30) of unexposed (control) females laid eggs. Adult emergence rates from larvae that were introduced into the cups differed significantly (Table 4). It was 17 times less likely for a larva to emerge when it was introduced into water in which PPF exposed female had laid eggs than when introduced into a cup in which unexposed female had laid eggs (Table 4), confirming that PPF was transferred.

**Table 4:** Adult emergence rate of late instar larvae introduced into oviposition cups in which *Anopheles* females laid eggs

	Mean proportion emergence (95% CI)	Odds ratio (95% CI)	*p*-value
Control - unexposed females	0.93 (0.89–0.97)	1	
Test - PPF-exposed females	0.45 (0.39–0.51)	0.06 (0.03–0.10)	0.007

Based on the control emergence, the corrected percent emergence inhibition observed was 52% (95% CI 46–56%). In other words, an individual female transferred the concentration of PPF to 100 ml of water that inhibited emergence of approximately 50% of larvae (EI_50_).

Chemical analysis showed that it was impossible to detect the PPF that was washed off the body of a single mosquito. Therefore, samples from 20 individuals were pooled for analysis on the LC-MS system. In total, PPF was washed off 140 individuals that had made contact with PPF (tests) and a similar number that was unexposed (controls). Consequently, there were seven pools of females in tests and in controls. No PPF was detected in any of the washes from control mosquitoes. PPF was below the detection limit in two of the test pools. The estimated amount of PPF washed off an individual female from the remaining five test pools was 141 μg, 120 μg, 93 μg, 117 μg and 89 μg. This results in an average of 112 μg (95% CI 103-123 μg) PPF washed off an individual mosquito. However, this is likely to be an overestimate since PPF levels were below detection limits in two sample pools which were not included in calculating this average. Assuming that an individual female transfers this entire amount of PPF to oviposition cups with 100 ml of water, we would expect a concentration of 1.12 mg PPF/L (1.12 ppm).

PPF was detected in three of the six test water samples but never in the control water samples. The estimated concentration of PPF in the individual water samples in the three positive pools were 330 ng/L, 160 ng/L and 190 ng/L. Therefore, the average estimated concentration of PPF in a single oviposition cup was approximately 230 ng/L (95% CI 180–290 ng/L); which is equivalent to 0.00023 mg/L (0.00023 ppm). Similarly, this is likely to be an overestimate since in three of the test water samples PPF was below the detection limit. Based on the bioassay results, this is the concentration that provided around 50% emergence inhibition of introduced larvae.

## Discussion

To our knowledge this is the first study to have developed a prototype bait-station for gravid *An. gambiae s.s*. for the auto-dissemination of PPF to aquatic habitats. We show in principle that gravid females can be attracted to a target, successfully pick up PPF and consequently transfer it to an aquatic habitat while laying eggs, thereby killing immature mosquito stages. Even though 200 gravid females were released in a relatively small space of approximately 170 m^3^, adult emergence from larval habitats was only inhibited by around 70% from ponds located less than 5 m from the bait-station but not from ponds 10 m away. These results strongly suggest that even if females can be lured successfully to a bait-station, they are most likely to transfer the PPF only to the closest available and detectable oviposition habitats.

This study highlights a number of challenges that need to be taken into consideration for the development of an efficient auto-dissemination approach for African malaria vectors. Based on previous work we consider exposing gravid females to PPF to be the most effective way to ensure transfer of the insecticide to an aquatic habitat [35]. This is because exposure of the female mosquito to PPF earlier in her life has significant impact on her fertility and fecundity [33, 34, 36, 46], lowering the females predisposition to visit an aquatic habitat. Attracting the gravid female is however more challenging than attracting a host-seeking or resting female, due to the scarcity of synthetic oviposition attractants [38, 47, 48].

Our study confirms previous findings that the chemical compound, cedrol [40], attracts gravid *An. gambiae s.s.* and might be used in a novel attract (−release) -and-kill approach. However, contrary to earlier findings by Lindh et al. [40] where twice as many gravid *An. gambiae s.l.* were recovered with 5 ppm cedrol than with water alone, we only achieved the same attractiveness with 20 ppm cedrol. This difference is likely due to the absence of directional air movement generated by the bait-station in our study. Lindh et al. [40] used modified BG-Sentinel traps that produce an air circulation with the help of a fan which likely led to the release of a larger amount of cedrol and water vapour, providing a stronger signal for oviposition site-seeking females. The absence of emergence inhibition from the ponds furthest away from the bait-station might not only indicate that gravid females flew the shortest distance from the bait-station to oviposit, but is likely also an indicator of the moderate attractiveness of the bait-station. Gravid females were released inside an experimental hut and assuming a random dispersal out through the open eaves, it is reasonable to assume, that a proportion of gravid females leaving the hut through the eave facing away from the bait-station went straight to the next open pond to lay eggs. To efficiently use a limited number of bait-stations in the natural field environment that is characterized with numerous aquatic habitats [18, 22], the attractiveness of bait-stations might be improved through innovative release technologies and formulation of more attractive chemical blends [47, 49–51].

Gravid females transferred more PPF from treated surfaces when PPF was applied as dust than when formulated in oil. There are two possible explanations for this. First, the oil might reduce the transfer of PPF to mosquitoes, with more of it adhering to the netting surface. Second, it might also be that the oil contributed to a larger proportion of PPF remaining on the mosquito’s body, thus limiting the chance of PPF getting in contact with water. Presumably, the hydrophobic oil attaches more strongly to the hydrophobic cuticle than to water [52]. In the approach used in this study, a lot of the active ingredient remained on the netting gauze and was wasted since not all the material was taken up. Furthermore, our chromatographic analyses reveal that an individual female transferred > 4800 times less PPF to the oviposition cup during egg-laying than the amount picked up from the treated netting. PPF on the insect cuticle is likely to decrease with time due to loss during flight, resting and penetration through the insect cuticle [53–55]. The chromatography analyses here confirm our findings from the bioassay, that a single female could transfer sufficient PPF to inhibit the emergence of 50% (EI50) of the larvae in 100 ml volumes of water. The average concentration of PPF detected in water used in the bioassays was 0.00023 mg/L (95% CI 0.000180–0.000290 mg/L), which correlates well with previously published results from laboratory assays when the EI_50_ was established at 0.000120 ng/L (95% CI 0.000090–0.000160 /L) [56]. The findings are also consistent with previous cage bioassays where females were contaminated in a plastic jar coated with PPF, and a single female caused approximately 50% of the introduced larvae not to develop into adult [35]. Taken together it appears that this is the average amount of our test formulation that a gravid *An. gambiae s.s.* can transfer to an aquatic habitat in a test system like ours. In our system, approximately 500 gravid females would be required to visit a water body 1 m^2^ in area and 10 cm deep, to transfer the optimal lethal concentration determined in the laboratory, which is highly unlikely under most natural conditions as gravid females comprise a very small proportion of the overall mosquito population. For large-scale application and cost-effective use of the relatively expensive active ingredient there is need to investigate strategies that use PPF more efficiently. Improved technologies such as electrostatic charging of PPF to enhance adherence of insecticide particles on insect body contact with contaminated surface and ensuring delivery of larger amounts of insecticide will be beneficial [49]. Whether this would improve the amount transferred to water or only increase the overall amount carried by the individual mosquito would need to be tested.

Mathematical models show that the success of auto-dissemination for malaria vector control is dependent on the abundance of adult vectors, the number and stability of larval habitats and persistence of the insecticide used [28, 29]. The auto-dissemination approach can be more appealing for controlling immature malaria vectors in aquatic habitats that are not easily accessible for insecticide application by teams on the ground [10]. This study highlights that the transfer of PPF to a larval habitat is dependent on the distance of the pond from the bait-station. Ponds closest to the bait-station are most likely to be visited consequently receiving sufficient amounts of PPF to have an impact on immature mosquito development. It is likely that vegetation cover, which was on purpose excluded from the experimental design might have further impacted on the ability of finding a suitable habitat and on the amount of PPF lost due to resting [55, 57]. Numerous bait-stations would therefore be required in the field for gravid mosquitoes to transfer sufficient lethal doses of PPF to their larval habitats. This is a substantial challenge considering the large number and extensive nature of water bodies utilized by African malaria vectors [18, 22].

Comparable semi-field studies to evaluate the possibility of auto-dissemination with PPF were done targeting host-seeking and resting *An. arabiensis* for PPF exposure using treated clay pots [30–32]. The efficiency of transfer reported from these studies were significantly higher, with an emergence inhibition of over 80% from the provided aquatic habitats. Factors that might explain the greater impacts in these studies include the use of numerous resting posts as dissemination stations for PPF and fewer, smaller-sized aquatic habitats. For instance, in two of these studies, the authors placed eight resting pots treated with PPF and provided only 2–4 small aquatic habitats of a 2.5 L capacity [30, 31]. Thus, the ratio of dissemination stations to oviposition habitats was 4:1 or 2:1 in these previous studies. This is in comparison to a dissemination station to oviposition habitats ratio of 1:3 in our study. Furthermore, in the previous studies with *An. arabiensis*, a very large number of 1500–5000 females were either introduced into the system, or reared inside the system [30–32], maximising the likelihood of a mosquito visiting a PPF-treated resting pot and the number of oviposition events in a single aquatic habitat.

A limitation of our study was the assessment of transfer of PPF by females by introducing insectary-reared late instar larvae into test ponds rather than monitoring adult emergence inhibition from larvae that hatch from the eggs laid by PPF-exposed females. This is likely to underestimate the impact of PPF disseminated by females into water bodies since it is expected that the impact on reducing adult emergence will be greater on immature stages that have prolonged exposure to the insecticide during larval development [58].

## Conclusion

Our study carried out under controlled conditions highlights potential limitations of the auto-dissemination strategy for the control of Afrotropical malaria vectors. Our results emphasize the need to investigate the required ratio of bait-stations to aquatic habitats for adult gravid females to transfer sufficient amounts of PPF for efficient control of immature malaria vectors in all aquatic habitats. The skip-oviposition behaviour recently observed in *An. gambiae s.s.* in cages [45] and in *An. arabiensis* in the field [59] is likely to benefit the auto-dissemination approach for malaria vector control since gravid females visit several habitats to lay eggs. Nevertheless, significantly more work is required in designing highly attractive bait-stations for gravid malaria vectors by identifying more attractants to compose highly attractive chemical blends, determining better mechanisms for optimum release of the attractants, identifying better and more cost-effective mechanisms for retaining and dispensing of PPF as well as improving the physical components of the bait-station to provide protective barriers from rain.

Additionally, insecticides of greater persistence in the environment than PPF, such as novaluron [26] might benefit the auto-dissemination approach for mosquito control. Most importantly, field evaluations are necessary to confirm the performance of such novel tools under natural conditions during both the dry and rainy seasons. Mosquitoes of other genera such as *Culex* might be used to amplify the transfer of PPF to larval habitats of *An. gambiae s.s*. [35], since these mosquitoes frequently share breeding habitats [21–23]. However, it might be challenging to attract this cohabiting species to the same bait-stations. In conclusion, the auto-dissemination strategy using PPF transferred by gravid malaria vectors requires significantly more research before it might be used operationally as a supplementary measure for malaria vector control.

## Data Availability

All data will be made available on reasonable request to the senior author.

## Abbreviations

HPLC: High performance liquid chromatography
*icipe*-TOC: International Centre of Insect Physiology and Ecology – Thomas Odhiambo Campus
LC/ESI-MS: Liquid chromatography/Electron spray ionization-Mass spectrometry
LC-MS: Liquid chromatography- Mass spectrometry
